# Editorial: Gut microbiota and immunity in health and disease: dysbiosis and eubiosis’s effects on the human body

**DOI:** 10.3389/fimmu.2024.1536258

**Published:** 2024-12-17

**Authors:** Veronica I. Dodero, Servaas A. Morré, Payam Behzadi

**Affiliations:** ^1^ Faculty of Chemistry, Bielefeld University Universitätsstr, Bielefeld, Germany; ^2^ Department of Genetics and Cell Biology, Institute of Public Health Genomics (IPHG), GROW Research Institute for Oncology and Reproduction, FHML, University of Maastricht, Maastricht, Netherlands; ^3^ Jacob Institute of Biotechnology and Bioengineering, Sam Higginbottom University of Agriculture, Technology and Sciences, Allahabad, UP, India; ^4^ Dutch Chlamydia trachomatis Reference Laboratory, Department of Medical Microbiology, Infectious Diseases and Infection Prevention, Care and Public Health Research Institute (CAPHRI), Maastricht University Medical Centre (MUMC+), Maastricht, Netherlands; ^5^ Department of Microbiology, Shahr-e-Qods Branch, Islamic Azad University, Tehran, Iran

**Keywords:** gut microbiota, innate immunity, adaptive immunity, health, disease, dysbiosis, eubiosis

The relationship between the gut microbiota and its human host is a complex and dynamic communication. Various host factors, such as genetics, immune function, age, gender, lifestyle (including pregnancy, delivery mode, nutrition, social behavior, and stress), body mass index (BMI), disease duration, and medical treatments, directly shape the composition of the gut microbiome (1–6).

When the gut microbiota is in balance—a state known as eubiotics—it supports the host’s health by producing beneficial microbial metabolites. On the other hand, an imbalance (dysbiosis), characterized by a dominance of harmful microbes and a lack of beneficial ones, can disrupt homeostasis and lead to various health problems (1, 7–9).

In recent years, research has intensified on the intricate connections between gut microbiota, eubiotics, and their impacts on human health and disease. To advance this growing field, this Research Topic of *Frontiers in Immunology* launched a dedicated Research Topic. We are proud to present a Research Topic of 11 impactful publications contributed by 69 researchers from around the globe.

In an experimental study conducted by Micek et al. in Poland, the researchers explored whether there is an association between the consumption of polyphenols, lignans, and herbal sterols and the presence of immune-stimulating microbiota, such as *Escherichia coli* and *Enterococcus* spp. The study included 95 non-obese participants aged 25–45 years, comprising 22 women and 73 men. The findings demonstrated a significant correlation between higher intake of these phytochemical compounds and a reduced risk of COVID-19 infection. The enhancement of gut microbiota likely mediated this effect. However, the authors recommended further research to confirm and expand upon these observations.

Another investigation, led by Chen et al., used a bidirectional two-sample Mendelian randomization approach to examine the causal relationship between nicotine dependence and gut microbiota composition. This study analyzed genome-wide association study (GWAS) data from 38,602 former smokers of African-American and European descent with varying levels of nicotine dependence. The findings suggested that the gut microbiome plays a role in nicotine metabolism and may influence disease progression associated with nicotine dependence.

These studies highlight the pivotal role of gut microbiota in modulating health outcomes and underscore the need for continued exploration in this dynamic research area.

In the review by Luo et al., the authors aimed to investigate the correlation between intestinal microbiota, vitamin A metabolism, and the retinoic acid (RA) signaling pathway in connection with bladder cancer. This review suggests intestinal microbiota may influence bladder tumorigenesis through the RA signaling pathway. Overall, the interaction between gut microbiota and RA exhibits synergistic anti-tumor effects.

Su et al. investigated the causal relationship between gut microbiota and six lung diseases: asthma, chronic bronchitis, chronic obstructive pulmonary disease (COPD), interstitial lung disease (ILD), lower respiratory tract infection (LRTI), and pulmonary arterial hypertension (PAH). The results revealed a correlation between the causality of gut microbiota and these lung diseases. Specifically, individual bacterial families may either increase or decrease the risk of developing lung diseases.

Li et al. conducted a Mendelian randomization study to investigate the causal relationship between gut microbiota composition, plasma metabolome, peripheral immune and blood cells, inflammatory cytokines, and obesity. Given that obesity is a metabolic and chronic inflammatory disease influenced by environmental and genetic factors, the researchers aimed to identify potential causal links between these factors. Among different correlations, the authors reported a pathway analysis that revealed 12 obesity-related metabolic pathways, particularly D-arginine, D-ornithine, linoleic acid, and glycerophospholipid metabolism, which were closely related to obesity.

Petakh et al. set out to explore how posttraumatic stress disorder (PTSD) affects gut microbiota and inflammatory biomarkers. By analyzing 15 studies, they uncovered significant shifts in the gut microbiota’s composition and diversity in people with PTSD. Interestingly, certain bacterial species seemed to play a role in these changes. However, when it came to inflammatory biomarkers, they didn’t find any notable differences between those with PTSD and those without it.

Masad et al. took a closer look at the effects of Manuka honey (MH) on colorectal cancer (CRC). Their research showed that MH, when taken orally, could trigger the interferon (IFN) signaling pathway through Toll-like Receptors (TLRs). This was observed in both BALB/c and C57BL/6 mouse models of CRC. Beyond that, MH seemed to reshape the tumor environment by boosting inflammatory cytokines and chemokines that regulate the immune response. The honey also influences gut microbiota, reducing harmful bacteria and enhancing its anti-tumor effects.

Warren et al. examined the microbiota-gut-brain-immune axis and its role in neuroinflammatory diseases. They argued that advancing global gut microbiome research and personalized healthcare means providing low- and middle-income countries (LMICs) with training, fostering collaboration, ensuring ethical engagement, and using standardized, multi-omics approaches.

In Warren et al.‘s second review, chronic stress, mental health issues, and immune dysfunction were explored as links to the microbiota-gut-brain axis. They reviewed evidence-based prevention strategies and potential therapeutic targets.

Hong et al. tackled the link between juvenile idiopathic arthritis and uveitis with gut microbiota using Mendelian randomization. Their findings suggested a direct relationship between changes in gut bacteria and the development of these conditions, offering new insight into their underlying causes.

Finally, Nenciarini et al. studied how *Saccharomyces cerevisiae* and *Lactobacillus* spp. interact to influence the immune system. Using strains from kefir, probiotics, and stool samples from a Crohn’s disease patient, they discovered that co-cultures of these microbes could activate immune cells and promote a tolerant immune response. These findings point out the potential of using microbial interactions to fine-tune immunity.

All in all: “Tell me what you eat, and I will tell you what you are.”

(https://courier.unesco.org/en/articles/tell-me-what-you-eat-and-ill-tell-you-who-you-are).
